# The association between women’s climate change anxiety, awareness of maternal–fetal health impacts, and their desire to avoid pregnancy

**DOI:** 10.1186/s12889-026-27173-y

**Published:** 2026-04-06

**Authors:** Songul Kekil, Sule Gokyildiz Surucu, Ayseren Cevik, Damla Kisrik, Ebru Gozuyesil, Burcu Avcibay Vurgec

**Affiliations:** 1 Midwifery Department, Faculty of Health Sciences, Gaziantep Islam Science and Technology University, Gaziantep, Türkiye; 2 Department of Midwifery, Student of Midwifery Doctorate Program, Cukurova University Institute of Health Sciences, Adana, Türkiye; 3 Midwifery Department, Faculty of Health Sciences, Cukurova University, Adana, Türkiye

**Keywords:** Pregnancy, Climate change, Awareness, Anxiety, Women

## Abstract

**Introduction:**

Climate change affects maternal and fetal health negatively. Climate change affects not only women’s efforts to protect their health but also their climate anxiety. Uncertainty about the future, in particular, could affect women’s choices to have children as well as their fertility intentions. This study aims to examine the associations between women's climate change anxiety, awareness of maternal–fetal health impacts of climate change, and their desire to avoid pregnancy.

**Methods:**

This study used a descriptive and cross-sectional design. It was conducted with 365 women who sought treatment in the family planning clinic affiliated with a maternity hospital between March 3 and May 30, 2025. Data were collected by meeting the participants face-to-face, using the Personal Information Form, the Desire to Avoid Pregnancy Scale, the Pregnancy-Specific Climate Change Awareness Scale, and the Climate Change Women's Anxiety Scale. The data obtained were analyzed using univariate and multivariate tests.

**Findings:**

The average age of women was 28.17 ± 4.30 years. It was found that 66.6% used family planning methods, 61.4% believed that climate change led to negative consequences, and 36.4% reportedly postponed pregnancy due to climate change. A positive relationship was found between avoiding pregnancy and awareness of climate change (*r* = 0.133, *p* = 0.011) and anxiety level (*r* = 0.201, *p* < 0.001).

**Conclusion:**

Increased climate anxiety in women was found to predict pregnancy avoidance. Although higher awareness of climate change effects on maternal and fetal health increased the desire to avoid pregnancy, it was not a determining factor.

## Introduction

Beyond being merely an environmental issue, climate change is considered a global crisis with significant effects on public health. Increases in global temperature, air pollution, and meteorological variables are associated with various adverse health outcomes, primarily cardiovascular, respiratory, and metabolic diseases [1–4]. The groups affected by these impacts the most include pregnant women, fetuses, and newborns. The developmental characteristics of organ systems and physiological adaptation processes make pregnancy and early life more sensitive periods to environmental stressors [5, 6]. Exposure related to climate change has been reported to be associated with obstetric outcomes such as low birth weight, preterm birth, and hypertensive pregnancy disorders [5–8]. Besides the physical health outcomes, research has been increasingly focusing also on the effects of climate change on mental health. Environmental uncertainty and perceived threats to the future are reported to be associated with stress, anxiety, and depressive symptoms [9–12]. In this regard, “climate anxiety” (eco-anxiety) is one of the concepts that stands out in the literature. Climate anxiety is defined as the experience of intense feelings of worry and uncertainty about the current and future potential consequences of climate change [13–15]. The future-oriented nature of climate anxiety has been reported to be associated with a reassessment of life plans, and in particular, decisions about having children in some individuals [16, 17].

The decision to have children or not is a contextual and multidimensional process that cannot be reduced to a single reason. In addition to economic conditions, career plans, and personal life goals, climate change is also considered a new source of uncertainty and risk perception added to this decision-making process [18, 19]. Concerns about the living conditions and safety of future children, in particular, have been reported to be associated with a reassessment of reproductive intentions [20, 21]. In line with this, individuals' awareness levels about the effects of climate change on maternal–fetal health involve the cognitive assessment of possible pregnancy outcomes. In this regard, while climate anxiety represents more of an emotional threat perception, awareness of maternal–fetal health reflects a cognitive dimension based on the assessment of pregnancy risks. The current literature have examined the relationships between these two constructs and the desire to avoid pregnancy separately, but studies evaluating them in tandem within the same model are limited in number [22, 23]. Women who visit family planning clinics constitute a group that actively makes reproductive decisions and seeks professional support about this issue, which makes them critically important in terms of examining this relationship. In this regard, this study examined the relationship between climate anxiety and awareness levels regarding the effects of climate change on maternal–fetal health and the desire to avoid pregnancy in women visiting family planning clinics. This study aims to contribute to the conceptual gap in the literature by evaluating the relationship between climate-related emotional and cognitive processes and the desire to avoid pregnancy within the same model.

### Research questions

1. What are women’s levels of awareness about the effects of climate change on maternal and fetal health?
2. What are women's levels of climate anxiety?
3. Does women’s climate anxiety affect their desire to avoid pregnancy?
4. Does women’s level of awareness about the effects of climate change on maternal and fetal health affect their desire to avoid pregnancy?

## Material and method

### Study design

The study used a descriptive and cross-sectional design, and was conducted between March 03 and May 30, 2025.

### Population and sample

The study was conducted in a province located in southern Türkiye. The population of the study consisted of women who sought treatment in the family planning outpatient clinic of a state hospital. The number of monthly treatment visits to this outpatient clinic is 1000 women. Since the data were collected in a six-month period, the population consisted of 6000 women on average. The study sample was calculated using the sample size calculator. With reference to the number of applications for six months (*N* = 6000), the minimum sample size was calculated as 362 with a 95% confidence interval and 5% margin of error, and 365 women participated in the study. While five women were excluded because they did not complete the questionnaires, eight women reportedly did not want to participate in the study.

The inclusion criteria were determined as being over 18 years of age, being literate, and having no communication difficulties. Women planning pregnancy within the next three months were excluded to maintain conceptual consistency with the Desire to Avoid Pregnancy (DAP) construct. Including these women could have introduced inconsistencies in measuring pregnancy avoidance attitudes. Therefore, this criterion was applied to preserve internal validity rather than to artificially affect DAP levels. The study population should be interpreted as women actively engaged in reproductive decision-making at the family planning clinic.

### Data collection tools

Data were collected by meeting the participants face-to-face, using the “Personal Information Form”, “Pregnancy-Specific Climate Change Awareness Scale” “Climate Change Women's Anxiety Scale” and “Desire to Avoid Pregnancy Scale”.

### Personal information form

The Personal Information Form is an interview form that was developed by the researcher based on the related literature. It consisted of 16 questions to collect data about the socio-demographic characteristics of participating women [24, 25].

### Pregnancy-Specific Climate Change Awareness Scale

The Pregnancy-Specific Climate Change Awareness Scale (PS-CCAS) was developed by Kısrık et al. [25]. The 21-item scale was designed to measure women’s awareness of the effects of climate change on maternal and fetal health. It comprises three subscales: *General*, *Maternal*, and *Fetal*. Each subscale score reflects awareness in that specific domain.

The total score ranges from 21 to 105, with higher scores indicating greater awareness of the effects of climate change among pregnant women. The overall Cronbach’s alpha for the scale was 0.946, while the reliability coefficients for the subscales were 0.871 for *General*, 0.924 for *Fetal*, and 0.907 for *Maternal*, indicating excellent internal consistency [25].

### Climate Change Women’s Anxiety Scale

The Climate Change Women’s Anxiety Scale (CCWAS) was developed by Süğüt et al. [26, 27]. It consists of 18 items, each rated on a five-point Likert scale: 1 = never, 2 = rarely, 3 = sometimes, 4 = usually, and 5 = always. The scale contains no reverse-scored items.

It includes three subscales: *Physiological Health* (items 1–8), *Behavior* (items 9–11, 13, 14, 17), and *Gender* (items 12, 15, 16, 18). Scores from each subscale reflect anxiety levels related to that specific domain. The total score ranges from 18 to 90, with higher scores indicating greater climate-related anxiety among women. The Cronbach’s alpha coefficient of the scale was reported as 0.969, demonstrating excellent internal consistency [26].

### Desire to Avoid Pregnancy Scale

The Desire to Avoid Pregnancy Scale (DAP) was developed by Rocca et al. [28] to prospectively assess women’s preferences regarding a potential future pregnancy and measure their desire to avoid pregnancy. The Turkish validity and reliability of the scale were conducted by Karataş Okyay et al. [29]. The scale includes 14 items, with the first five addressing feelings about becoming pregnant within the next three months and the remaining nine focusing on having a baby within the next year. It uses a five-point Likert scale ranging from 0 (“strongly agree”) to 4 (“strongly disagree”). Seven items (3, 7, 9, 11, 12, 13, 14) are scored reversely. After the relevant items are reversed, all are summed and divided by 14 to obtain a mean score. Higher total scores indicate a greater desire to avoid pregnancy [29].

### Data analysis

Data were analyzed using the SPSS 22 package program [30]. The study includes dependent and independent variables. While the participants’ descriptive and obstetric characteristics and their knowledge about climate change constitute the independent variable, their desire to avoid pregnancy, climate anxiety and climate awareness levels constitute the dependent variables. Normality distribution of the data were analyzed using the Kolmogorov–Smirnov test, indicating that the data showed non-parametric distribution (*p* ≤ 0.05). Frequency analyses were performed to determine the participants’ descriptive and obstetric characteristics, knowledge about climate change, and total scale scores. Spearman correlation was used to determine the relationship between the scales, and linear regression analysis was used to determine the factors affecting the desire to avoid pregnancy.

### Findings

The average age of the women was 28.17 ± 4.30 years. Of all the participants, 56.4% did not plan a pregnancy and 66.6% used family planning methods. Table 1 presents other descriptive and obstetric characteristics of the participants.

**Table 1:** Findings of the participants’ descriptive and obstetric characteristics

	n (%)
Age	
≤ 28	188 (51.5)
> 28	177 (48.5)
Education level	
Primary school	102 (27.9)
Secondary school	95 (26)
High school	131 (35.9)
University and above	37 (10.2)
Partner’s education level	
Primary school	93 (25.5)
Secondary school	88 (24)
High school	144 (39.5)
University and above	40 (11)
Income level	
Income less than expenses	241 (66)
Income equal to expenses	111 (30.4)
Income more than expenses	13 (3.6)
Family type	
Nuclear family	316 (86.6)
Extended family	49 (13.4)
Chronic diseases	
Yes	49 (13.4)
No	316 (86.6)
Duration of marriage	
0–5 years	162 (44.4)
6–10 years	115 (31.5)
11 years and over	88 (24.1)
Using a family planning method	
Yes	245 (66.6)
No	120 (33.4)
Planning a pregnancy	
Do not want a pregnancy	206 (56.4)
1 year	39 (10.7)
2 years	53 (14.5)
≥ 3 years	67 (18.4)

Frequency

It was found that 49% of the women were partially informed about climate change, 38.1% followed the news about climate change, 61.4% thought that climate change affected all people negatively and 67.9% thought that it affected themselves negatively, 51.2% thought that climate change prevented pregnancy planning, and 36.4% reportedly postponed pregnancy due to climate change (Table 2).

**Table 2:** Findings of women’s knowledge about climate change and thoughts about the effects of climate change

	**n (%)**
Knowledge about climate change	
Yes	52 (14.2)
Partial	179 (49)
No	134 (36.8)
Thoughts about the effects of climate change on humanity	
Positive	30 (5.5)
Negative	224 (61.4)
Don’t know	121 (33.2)
Following news about climate change	
Yes	139 (38.1)
No	226 (61.9)
Individual effects of climate change	
Positive	66 (18.1)
Negative	248 (67.9)
No effect	51 (14)
Effect of climate change on avoiding pregnancy	
Yes	187 (51.2)
No	178 (48.8)
Postponing pregnancy due to climate change	
Yes	133 (36.4)
No	232 (63.6)

Frequency

Women’s Desire to Avoid Pregnancy Scale, Climate Anxiety Scale and Climate Awareness Scale scores were determined 2.57 ± 0.60, 59.35 ± 13.96 and 70.17 ± 16.79, respectively (Table 3).

**Table 3:** Findings of women’s desire to avoid pregnancy, climate anxiety and climate awareness scales

	X̄ ± SD	Cronbach’s alpha
Desire to Avoid Pregnancy Scale	2.57 ± 0.60	0.76
Climate Anxiety Scale	59.35 ± 13.96	0.90
Physiological	26.11 ± 7.14	0.89
Behavioral	19.63 ± 5.44	0.74
Gender	13.60 ± 4.02	0.66
Climate Awareness Scale	70.17 ± 16.79	0.88
General	19.97 ± 6.36	0.67
Fetal	25.44 ± 7.29	0.77
Maternal	24.75 ± 6.21	0.88

Frequency

A weak positive correlation (*r* = 0.201, *p* < 0.001) was found between the Desire to Avoid Pregnancy Scale score and the Climate Anxiety Scale score, and a weak positive correlation was detected between the Pregnancy-Specific Climate Change Awareness Scale score (*r* = 0.133, *p* = 0.011) (Table 4).

**Table 4:** Scale correlations

	**DAP**		**CCWAS**		**PS-CCAS**	
	**r**	***p***	**r**	***p***	**r**	***p***
DAP	1.000	-	0.201^**^	< 0.001	0.133^*^	0.011
CCWAS			1.000	-	0.846^**^	< 0.001
PS-CCAS					1.000	-

*DAP* The Desire to Avoid Pregnancy Scale, *CCWAS* The Climate Change Women’s Anxiety Scale, *PS-CCAS* The Pregnancy-Specific Climate Change Awareness Scale

Spearmen korelasyon, ^**^Correlation is significant at the 0.01 level (2-tailed), ^*^Correlation is significant at the 0.05 level (2-tailed)

Regression analysis was performed to determine the predictor variables affecting the Desire to Avoid Pregnancy Scale scores. Climate anxiety level was found to be a predictive factor for Desire to Avoid Pregnancy (R^2^ = 0.083, *p* = 0.009) (Table 5). Increased climate anxiety was found to have an increasing effect on the desire to avoid pregnancy. On the other hand, although increased awareness of the effects of climate change on maternal and fetal health increased the desire to avoid pregnancy, it was not a determining factor (*p >* 0.05) (Table 5). Age, education level, income level, duration of marriage, and use of family planning were included as control variables in the regression analysis. None of these variables showed a statistically significant association with the desire to avoid pregnancy (*p >* 0.05).

**Table 5:** Factors affecting desire to avoid pregnancy

The Desire to Avoid Pregnancy Scale	B	S.E	β	t	*p*	R	*R*^2^
Constant	1.809	0.320		5.654	< 0.001	0.288	0.083
CCWAS	0.011	0.004	0.252	2.611	0.009		
PS-CCAS	−0.003	0.003	−0.084	−0.877	0.381		

Linear regresyon, *DAP* The Desire to Avoid Pregnancy Scale, *CCWAS* The Climate Change Women’s Anxiety Scale, *PS-CCAS* The Pregnancy-Specific Climate Change Awareness Scale

## Discussion

This study aimed to examine the associations between women's climate change anxiety, awareness of the maternal–fetal health impacts of climate change, and their desire to avoid pregnancy. An analysis of the literature indicates no studies that determined women's awareness of climate change anxiety and its effects on maternal and fetal health and their desire to avoid pregnancy in tandem. Therefore, this study is considered to be a pioneering study that could contribute to the literature.

Around half of the women participating in this study reported that they had partial knowledge about climate change, one-third followed the news on this issue, and more than half reported that climate change negatively affected human health and women’s health. The related literature reports that women have similar amount of knowledge about climate change, they follow news about climate change, and climate change affects both human health and women’s health [27, 31–33].

 This study found that 51.2% of the participants believed that climate change prevented pregnancy planning. No studies in the literature were found to have directly evaluated women's views on climate change and family planning. However, studies have reported that women are concerned about the future of their children to be born due to the effects of climate change; that their environmental uncertainties were associated with lower fertility intentions; and that climate change is an important reason not to have children [18, 34]. In their study on ecological-reproductive concerns in the era of climate change, Schneider-Mayerson and Leong [18] reported that women's higher levels of anxiety were associated with lower fertility desires and concerns about climate change [18].

This study found that women had a high level of climate change anxiety. An analysis of the literature indicates only one study that analyzed the level of anxiety directly related to women’s health. Hamlaci et al. (2024) examined the effect of anxiety level related to climate change on the use of feminine hygiene products and found a high level of anxiety in women [35]. Studies conducted using relevant measurement tools such as the Climate Change Anxiety Scale [36], the Climate Change Worry Scale [37], and the Eco-Anxiety Scale [38] reported low levels of climate change anxiety in women [39–42]. This difference may be caused by the measurement tools used to assess different parameters or the different populations studied.

This study found that women had a high level of awareness about the effects of climate change on the pregnancy process. An analysis of the literature indicated no studies that analyzed the effects of climate change awareness on the pregnancy process. Topsakal and Çevik [43] examined the effects of climate change awareness on fertility desire and reported that women had a high level of climate change awareness [43]. Other studies similarly reported that women had a high level of climate change awareness [44–46]. In this study, women’s high level of awareness about the effects of climate change on the pregnancy process may be related to the fact that women follow the news on the subject and have high awareness because they live in a region where the effects of climate change are felt.

Climate anxiety is known to be more common among individuals who take environmental problems more seriously and experience the effects of climate change [36]. Besides, women have been reported to have higher climate change anxiety than men [47, 48, (Closson K, Card KG, Logi C, Aran N, Sachal AS, Bratu A, et al: Gender differences in climate change anxiety, unpublished)]. It is reported that women's psychological well-being and mental health may be affected both directly and indirectly due to high levels of anxiety and stress and high levels of awareness. This may be associated with lower fertility intentions. This study found that the desire to avoid pregnancy increased as the level of climate change awareness increased. Although the literature includes a limited number of studies on this subject, women who have knowledge about climate change were reported to have a decreased desire for fertility [32, 43]. Therefore, awareness of climate change may be associated with higher levels of anxiety. Our findings are in line with the literature in this respect.

This study found that 36.4% of the women postponed pregnancy due to climate change, 56.4% did not plan pregnancy and 66.6% used family planning methods, and the desire to avoid pregnancy was found to increase as the level of anxiety about climate change increased. An analysis of the literature indicates that individuals' concerns about the future of the children they have or plan to have and their high level of anxiety about climate change decrease their intention to have children significantly [18, 34]. Helm et al. [19] reported that women of reproductive age who experience climate change anxiety feel guilt and anxiety about their decision to expose their children to a threatened future [19]. The literature includes a limited number of studies on this issue, yet it was reported that women who were informed about climate change had decreased fertility desire [18, 34].

This study found that the climate anxiety level was a predictive factor for the desire to avoid pregnancy. The literature was found to include no studies on this issue. However, studies examining the relationship between climate change anxiety and fertility desire reported a significant relationship between climate anxiety and the desire to avoid pregnancy [42, 49].

This study has several strengths and limitations. The study is particularly valuable as the first study to examine how women’s climate anxiety and awareness of the impacts of climate change on maternal and fetal health influence their reproductive intentions. Additionally, conducting the study in a region highly affected by climate change enhances its relevance. However, the findings are limited in their generalizability because the study was carried out in a single hospital-based family planning clinic and included only women who were not planning a pregnancy and were actively seeking healthcare services.

Considering these limitations, future research would benefit from involving larger and more diverse populations, preferably through community-based studies. Furthermore, comprehensive studies employing qualitative research methods are warranted to gain a deeper understanding of women’s perceptions, experiences, and decision-making processes concerning climate change and reproductive intentions. Such efforts could provide more nuanced insights and inform public health strategies aimed at addressing climate-related reproductive concerns.

## Conclusion

This study found that women have high levels of awareness about the effects of climate change on the pregnancy process and high levels of climate anxiety. The results of the study indicate a weak relationship between climate anxiety and the desire to avoid pregnancy. On the other hand, while the level of awareness regarding maternal–fetal health was found to be associated with the desire to avoid pregnancy, it was not an independent predictor in the multivariate model. A significant proportion of women believe that climate change could negatively impact their health and the future of their potential children, which suggests that the climate crisis has a psychosocial dimension in the context of reproductive decisions. These findings indicate that the decision to have children may be shaped not only by individual or economic factors, but also by environmental uncertainties and concerns about the future.

This study contributes to the examination of the relationship between climate concerns and reproductive decisions. Future studies may explore the direction of this relationship over time more clearly. Besides, it is important to evaluate the effects of counseling and educational interventions on women's climate-related concerns and reproductive decision-making processes. In reproductive health services, considering women’s perceptions and concerns about climate change may contribute to a more comprehensive understanding of decision-making processes.

## Data Availability

The data of the research is stored in an external hard-disk and the first author's PC. Data are available on request.
